# Efficacy and safety of omadacycline for treating complicated skin and soft tissue infections: a meta-analysis of randomized controlled trials

**DOI:** 10.1186/s12879-024-09097-3

**Published:** 2024-02-19

**Authors:** Wenxin Liang, Hong Yin, Huiling Chen, Juan Xu, Yun Cai

**Affiliations:** 1 Center of Medicine Clinical Research, Department of Pharmacy, Medical Supplies Center, Chinese PLA General Hospital, Beijing, 100853 People’s Republic of China; 2 Department of Pharmacy, Medical Supplies Center, Chinese PLA General Hospital, Beijing, 100853 People’s Republic of China

**Keywords:** Omadacycline, Linezolid, Complicated skin and soft tissue infections, Aminomethylcycline

## Abstract

**Objective:**

In the present study, we aimed to compare the clinical efficacy and safety of omadacycline (OMC) with its comparators for the treatment of complicated skin and soft tissue infections (cSSTIs) in adult patients.

**Methods:**

Randomized controlled trials (RCTs) evaluating OMC for cSSTIs were searched in databases of PubMed, Embase, Cochrane, Web of Science, and Clinical Trial, up to July 2022. The primary outcomes were clinical efficacy and microbiological response, with secondary outcome was safety.

**Results:**

Four RCTs consisting of 1,757 patients were included, with linezolid (LZD) as a comparator drug. For clinical efficacy, OMC was not inferior to LZD in the modified intent-to-treat (MITT) (OR: 1.24, 95% Cl: [0.93, 1.66], *P* = 0.15) and clinically evaluable (CE) populations (OR: 1.92, 95% Cl: [0.94, 3.92], *P* = 0.07). For microbiological response, OMC was numerically higher than LZD in the microbiologically evaluable (ME) (OR: 1.74, 95% Cl: [0.81, 3.74], *P* = 0.16) and microbiological MITT (micro-MITT) populations (OR: 1.27, 95% Cl: [0.92, 1.76], *P* = 0.14). No significant difference was found in subpopulations of monomicrobial or polymicrobial mixed infection populations. The mortality and adverse event rates were similar between OMC and LZD.

**Conclusions:**

OMC was as good as LZD in terms of clinical efficacy and microbiological response, and has similar safety issues in treating cSSTIs. OMC might be a promising option for treating cSSTIs in adult patients.

## Background

Skin and soft tissue infections (SSTIs) are caused by bacteria invading the skin and surrounding tissues, which is a common problem in hospitals. In the United States, there are more than 14 million outpatients with SSTIs and almost 900,000 hospitalized patients yearly [1].

According to the extent of infected skin, the Infections Diseases Society of America (IDSA) divides SSTIs into complicated SSTIs (cSSTIs) and uncomplicated SSTIs [2]. cSSTIs include deep soft tissue infections, such as necrotizing infections, infected ulcers, infected burns, and severe abscesses [3]. Uncomplicated SSTIs refer to superficial infections, including cellulitis, simple abscesses, impetigo, and furuncles [3]. Gram-positive bacteria are the primary pathogens of cSSTIs, among which *Staphylococcus aureus* is the most common one, and methicillin-resistant *Staphylococcus aureus* (MRSA) accounts for 46% of *S. aureus* isolates [4]. Other Gram-positive bacteria include *Streptococcus pyogenes*, *Enterococcus faecalis*, etc. In contrast, Gram-negative bacteria are less common in cSSTIs [4].

The most recent guideline of the Surgical Infection Society (SIS) on the management of cSSTIs suggests that vancomycin, linezolid (LZD), daptomycin, ceftaroline, and telavancin are first-line agents for cSSTIs caused by MRSA [5]. Vancomycin has always been the standard treatment for MRSA-caused cSSTIs, while LZD has been proven to be an effective substitute for vancomycin [6]. However, these drugs have limitations in their clinical application. Vancomycin has low tissue penetration and nephrotoxicity risk during treatment. Moreover, only intravenous dosage forms of vancomycin can be used. Long-term use of LZD may cause thrombocytopenia, as well as peripheral and central neuropathies [7, 8]. Since cSSTIs are characterized by deep tissue involvement and diverse pathogens, long-term antimicrobial therapy is usually required. Therefore, the optimal antibiotics for cSSTIs must have good tissue distribution, a broad antibacterial spectrum, and long-term medication safety and compliance.

Omadacycline (Nuzyra, PTK 0796, OMC) is a third-generation tetracycline derivative, but the first aminomethylcycline. It inhibits the synthesis of bacterial proteins by binding to 30 s ribosomal subunits and blocking the binding of aminoacyl tRNA [9, 10]. The structural modification of OMC makes it overcome the common tetracycline resistance mechanisms, such as the increasing number of efflux pumps and the production of ribosomal protective proteins [11, 12]. OMC has been proven to have good antibacterial activity against common clinical Gram-positive bacteria, Gram-negative bacteria, and anaerobes, showing low minimum inhibitory concentrations [13, 14]. OMC has high oral bioavailability, allowing intravenous injection and oral administration [15], which is convenient for outpatients and discharged patients with medicine. The dosage of once a day improves the patient's compliance with medication [16]. In addition, OMC has the characteristics of a large steady-state distribution volume and a high tissue penetration rate [10, 17, 18]. For patients with liver and kidney impairments, there is no need to adjust the dosage of OMC [19]. The FDA approved OMC to treat acute bacterial skin and skin structure infections and community-acquired bacterial pneumonia in October 2018 [20].

The present meta-analysis included all available randomized controlled trials (RCTs) to comprehensively evaluate the efficacy and safety of OMC in the treatment of cSSTIs. Collectively, our current findings provided valuable insights into the treatment for cSSTIs in clinical practice.

## Methods

### Study search and selection

This study was conducted in accordance with the Preferred Reporting Program for Systematic Review and Meta-Analysis (PRISMA) statement [21]. PubMed, Embase, Cochrane, Web of Science, and Clinical Trial databases were searched from the establishment of the database to July 2022, regardless of language, using the following search terms: “omadacycline” OR “nuzyra” OR “PTK 0796”. Duplicate records were eliminated using Endnote X9, and two reviewers (Liang and Yin) independently monitored the records to avoid bias according to the inclusion and exclusion criteria. If any disagreement arose during the review process, a third reviewer (Xu) would decide. Inclusion criteria were set as follows: ① RCTs of efficacy and safety, ② patients over 18 years old with SSTIs, and ③ the patients in the test group were treated with OMC, and the patients in the control group were treated with other drugs. Exclusion criteria were set as follows: ① republished literature, ② review, case report, etc., and ③ data from the same RCT.

### Data extraction and quality assessment

In all included studies, the data were extracted primarily by two researchers independently, and if there was disagreement during extraction, it was examined and determined by a third investigator. The following information was extracted from included studies, such as the first author, publication year, research place, start and end time, intervention measures, sample size, outcome indicators, pathogenic microorganisms, infection types, size of lesion and drainage procedures. According to the items in the Cochrane Collaboration Risk of Bias Tool, the risk of bias in all included studies was rated as “low risk”, “unclear”, or “high risk” [22]. The quality of the included studies was evaluated using the Jadad scale [23]. A total score of 0 ~ 5 points, including 0 ~ 2 for randomization, 0 ~ 2 for blinding, and 0 ~ 1 for withdrawal.

### Definitions

The modified intent-to-treat (MITT) population included all randomized patients who did not have a sole Gram-negative causative pathogen at baseline. The clinically evaluable (CE) population included patients in the MITT population who had a qualifying infection as per study-entry criteria, received the study drug, did not take other antibiotics that may confound with results, and had an assessment of outcome during the protocol-defined window.

The microbiologically evaluable (ME) population consisted of the CE population who had at least one Gram-positive pathogen at baseline. The microbiological MITT (micro-MITT) population was composed of MITT patients with at least one Gram-positive bacterial pathogen identified from blood culture or the sample obtained from the cSSTI site at baseline.

### Outcome measurement

Clinical efficacy endpoints were defined as the infection was fully resolved, and no further antimicrobial treatment was required at the end of treatment and post-treatment evaluation (PTE) in the MITT and CE populations [24]. The microbiological response was determined in the ME and micro-MITT population as eradication (absence of original baseline pathogen) or presumed eradication (no source specimen to culture in a subject assessed with a clinical success) of baseline pathogens [24]. Adverse events (AEs) were defined as AEs that emerged during or after administration, increased in severity, or were associated with the study drug during the study period.

### Data analysis

Review Manager 5.3 software was used for meta-analysis. Qualitative data were expressed by odds ratio (OR) and its 95% Cl, and quantitative data were described by mean difference (MD) and its 95% Cl. The heterogeneity test was evaluated by the Cochrane *I*^*2*^ statistics. When P < 0.10 or *I*^*2*^ > 50%, it was considered that there was statistical heterogeneity among the studies, and the random effects model was used for meta-analysis; otherwise, the fixed effects model was used. P < 0.05 was considered statistically significant.

This meta-analysis was registered in the International Prospective Register of Systematic Reviews (PROSPERO: CRD 42022362152).

## Results

### Study selection and characteristics

**Fig. 1:**
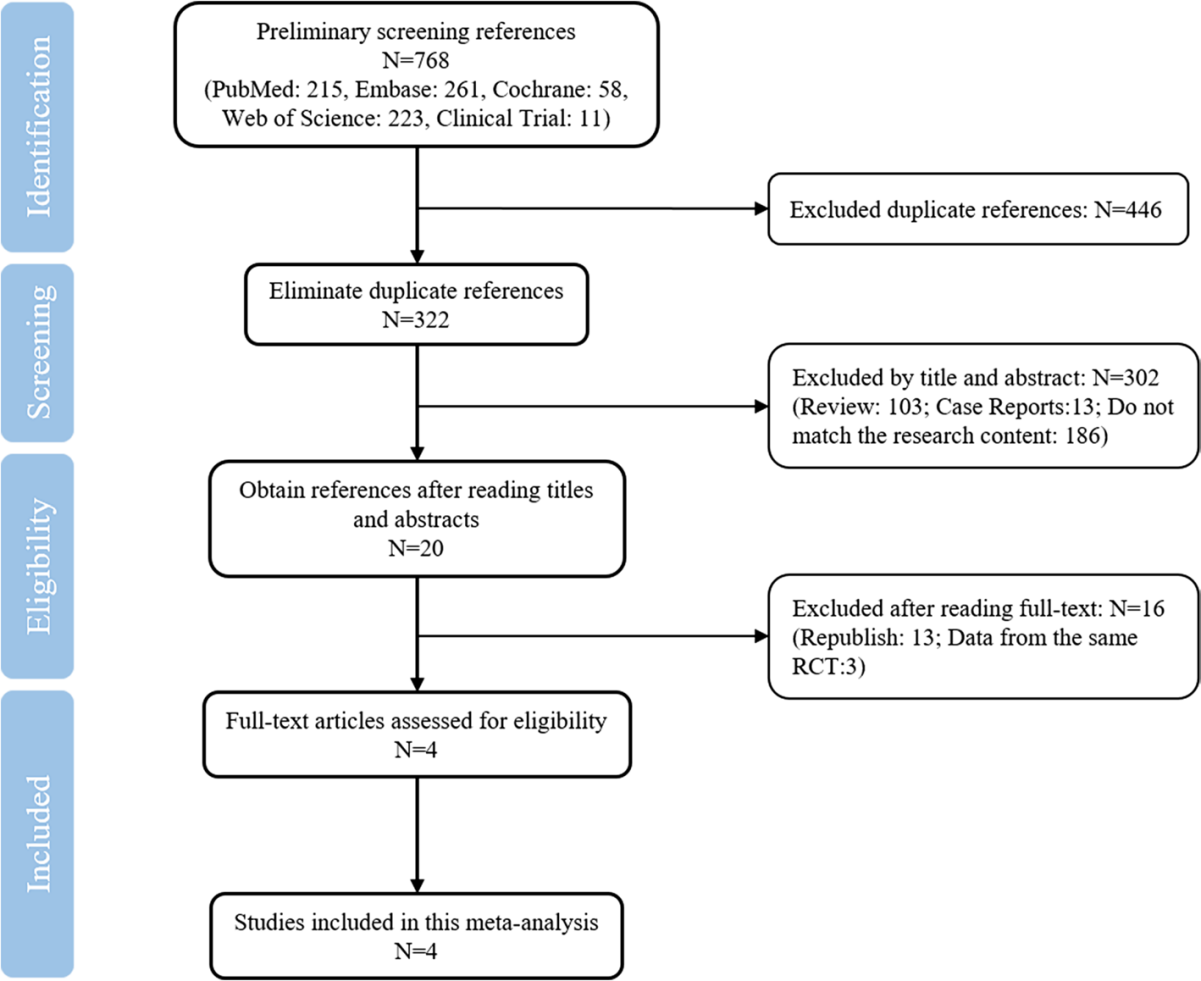
Flow chart of reference screening

**Fig. 2:**
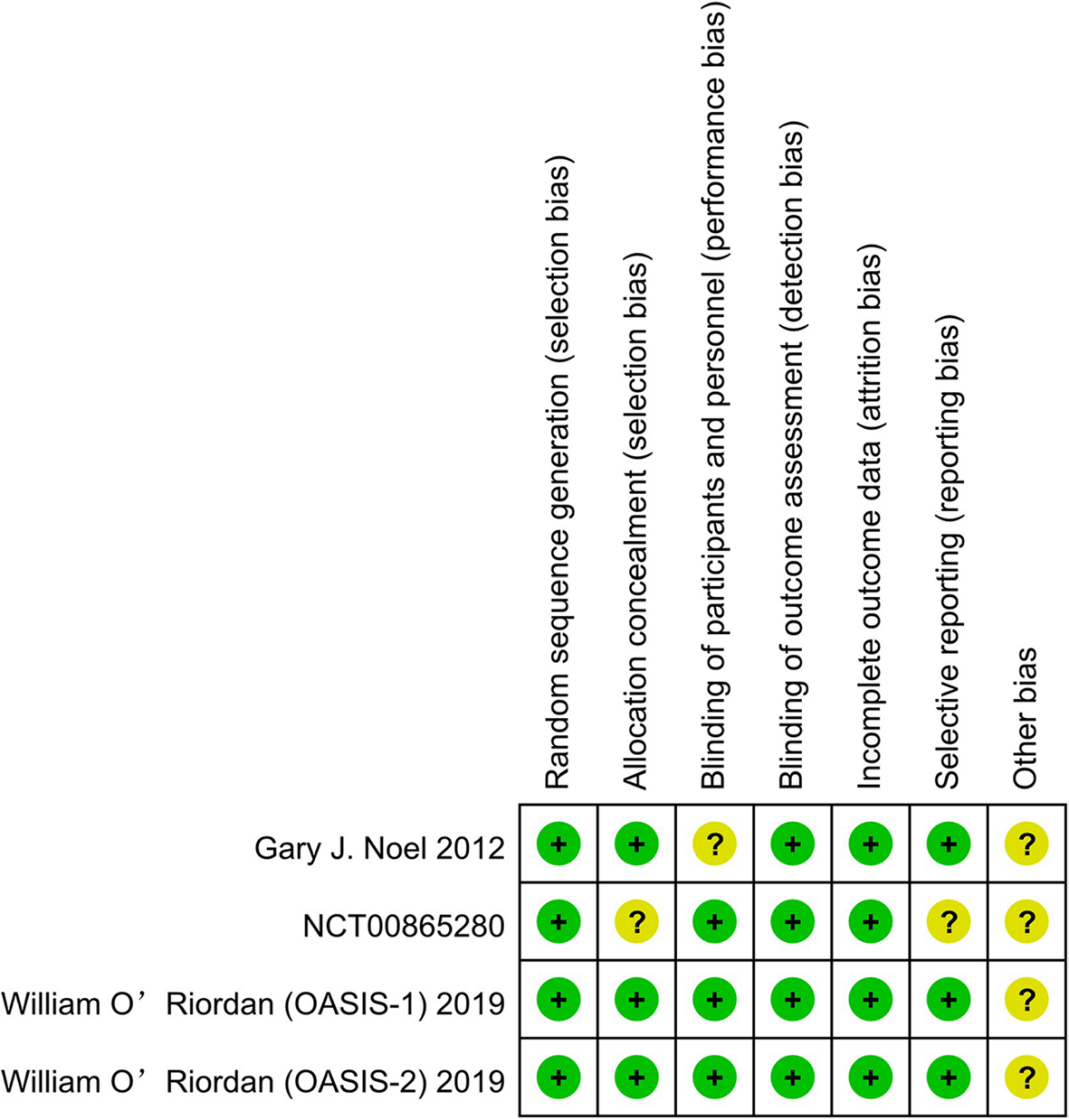
Summary graph of the risk of bias

A total of 768 references were obtained (PubMed: 215, Embase: 261, Cochrane: 58, Web of Science: 223, Clinical Trial: 11), while 446 duplicates were excluded. By reading topics and abstracts, 302 references were excluded. Finally, 20 studies remained. By reading the full text, four RCTs [25–28] consisting of 1,757 patients were included. Figure 1 illustrates the process of literature search and screening. Table 1 shows the essential characteristics, and all compared drugs included in this meta-analysis were LZD. Moreover, all included studies were high-quality RCTs, and Fig. 2 shows the risk chart of bias.

**Table 1:** Basic information of included studies

No	Author	Year	Sites	Start and End	Treatment Plan		Subjects		Pathogen Detected at Baseline	
					OMC	LZD	OMC	LZD	OMC	LZD
1	Gary J. Noel	2012	11 sites in the USA	2007.07–2008.01	100 mg (i.v.) q24h/200 mg (p.o.) q24h	^a^600 mg (i.v.) q12h/600 mg (p.o.) q12h	118	116	G^+^ pathogen: *S. aureus*: 72 MRSA: 44 MSSA:\ G^+^ bacterium other than *S. aureus*: 3	G^+^ Pathogen: *S. aureus*: 55 MRSA: 32 MSSA:\ G^+^ bacterium other than *S. aureus*: 7
2	NCT00865280	\	7 sites in the USA	2009.04–2010.04	100 mg (i.v.) q24h/300 mg (p.o.) q24h	^b^600 mg (i.v.) q12h/600 mg (p.o.) q12h	70	73	\	\
3	William O’Riordan (OASIS-1)	2019	55 sites in the USA, Peru, South Africa, and Europe	2015.06–2016.05	100 mg (i.v.) q12h for 1 day + 100 mg (i.v.) q24h for 2 days + 100 mg (i.v.) or 300 mg (p.o.) q24h	600 mg (i.v.) q12h for 3 days + 600 mg (i.v.) or 600 mg (p.o.) q12h	323	322	Mono-G^+^: 156 Poly-G^+^: 31 Mixed: 41 G^+^ Pathogen: *S. aureus*: 156 MRSA: 69 MSSA: 88 *S. pyogenes*: 11 *Streptococcus anginosis* group: 47 *E. faecalis*: 10	Mono-G^+^: 171 Poly-G^+^: 27 Mixed: 29 G^+^ Pathogen: *S. aureus*: 151 MRSA: 50 MSSA: 102 *S. pyogenes*: 18 *Streptococcus anginosis* group: 37 *E. faecalis*: 13
4	William O’Riordan (OASIS-2)	2019	33 sites in the USA	2016.08–2017.06	450 mg (p.o.) q24h for 2 days + 300 mg (p.o.) q24h	600 mg (p.o.) q12h for 2 days + 600 mg (p.o.) q12h	368	367	Mono-G^+^: 184 Poly-G^+^: 60 Mixed: 32 G^+^ Pathogen: *S. aureus*: 220 MRSA: 104 MSSA: 120 *S. pyogenes*: 29 *Streptococcus anginosis* group: 57 *E. faecalis*: 7	Mono-G^+^: 212 Poly-G^+^: 37 Mixed: 38 G^+^ Pathogen: *S. aureus*: 233 MRSA: 107 MSSA: 130 *S. pyogenes*: 16 *Streptococcus anginosis* group: 45 *E. faecalis*: 10
**No**	**Author**	**Note**								**Quality score**
		**Infection types**				**Size of lesion**		**Drainage Procedures**		
		Cellulitis/Erysipelas		Major abscess	Wound infection					
1	Gary J. Noel	^c^OMC 8 (7%) LZD 10 (9%)		^c^OMC 73 (66%) LZD 72 (67%)	^c^OMC 21 (19%) LZD 17 (16%)	^g^Abscesses: OMC 12.6 cm; LZD 11.3 cm Wound infection: 13.6 cm		Incision and drainage of the infected site in all abscess patients is permitted before or within 24 h after treatment		4
2	NCT00865280	^d^OMC 39 (57%) LZD 45 (63%)		\	^d^OMC 13 (19%) LZD 13 (18%)	\		\		3
3	William O’Riordan (OASIS-1)	^e^OMC 123 (39%) LZD 118 (38%)		^e^OMC 91 (29%) LZD 89 (29%)	^e^OMC 102 (32%) LZD 104 (33%)	^h^OMC 299.5 (77 ~ 4100) cm^2^; LZD 315.0 (88 ~ 6739) cm^2^		Patients with major abscesses had allowed drainage procedures before (or within 48 h following) the first dose		5
4	William O’Riordan (OASIS-2)	^f^OMC 86 (24%) LZD 84 (23%)		^f^OMC 64 (18%) LZD 62 (17%)	^f^OMC 210 (58%) LZD 214 (59%)	^h^OMC 322 (198 ~ 495) cm^2^; LZD 294 (190 ~ 462) cm^2^		Patients with major abscesses had allowed drainage procedures before (or within 48 h following) the first dose		5

*S. aureus Staphylococcus aureus*, *MRSA* meticillin-resistant *S. aureus*, *MSSA* meticillin-susceptible *S. aureus*, *S. pyogenes, Streptococcus pyogenes*, *Streptococcus anginosis* group: includes *S. anginosus*, *Streptococcus intermedius*, and *Streptococcus constellatus*, *E.faecalis Enterococcus faecalis*

Mono-G^+^: monomicrobial Gram-positive infection; Poly-G^+^: polymicrobial Gram-positive infection; Mixed: polymicrobial Gram-positive and Gram-negative infection

^a^with or without aztreonam

^b-^with moxifloxacin

^c^in intent-to-treat population, *n* = 111 and 108

^d^in clinically evaluable population, *n* = 68 and 72

^e^in modified intent-to-treat population, *n* = 316 and 311

^f^in modified intent-to-treat population, *n* = 360 and 360

^g^mean maximum dimension

^h^mean maximal linear dimension

### Clinical efficacy

**Fig. 3:**
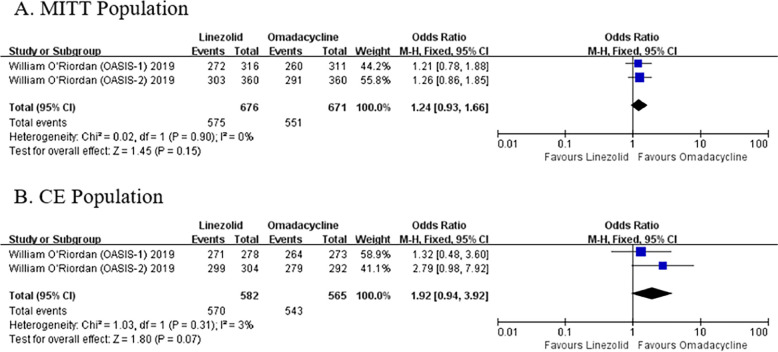
Clinical efficacy rates of OMC and LZD in the MITT and CE populations

The results of the meta-analysis showed that the efficacy of OMC was not inferior to LZD in both the MITT population (OR: 1.24, 95% Cl: [0.93, 1.66], *P* = 0.15) and CE population (OR: 1.92, 95% Cl: [0.94, 3.92], *P* = 0.07) (Fig. 3).

### Microbiological response

**Fig. 4:**
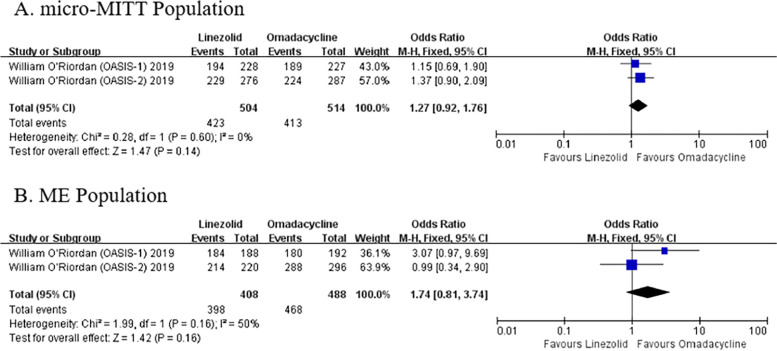
Microbiological response of OMC and LZD in the micro-MITT and ME populations

Two studies reported microbiological responses in the micro-MITT and ME populations under the same baseline. Generally, the microbiological response of the OMC group was numerically higher compared with the LZD group (micro-MITT population: OR: 1.27, 95% Cl: [0.92, 1.76], *P* = 0.14; ME population: OR: 1.74, 95% Cl: [0.81, 3.74], *P* = 0.16) (Fig. 4).

**Fig. 5:**
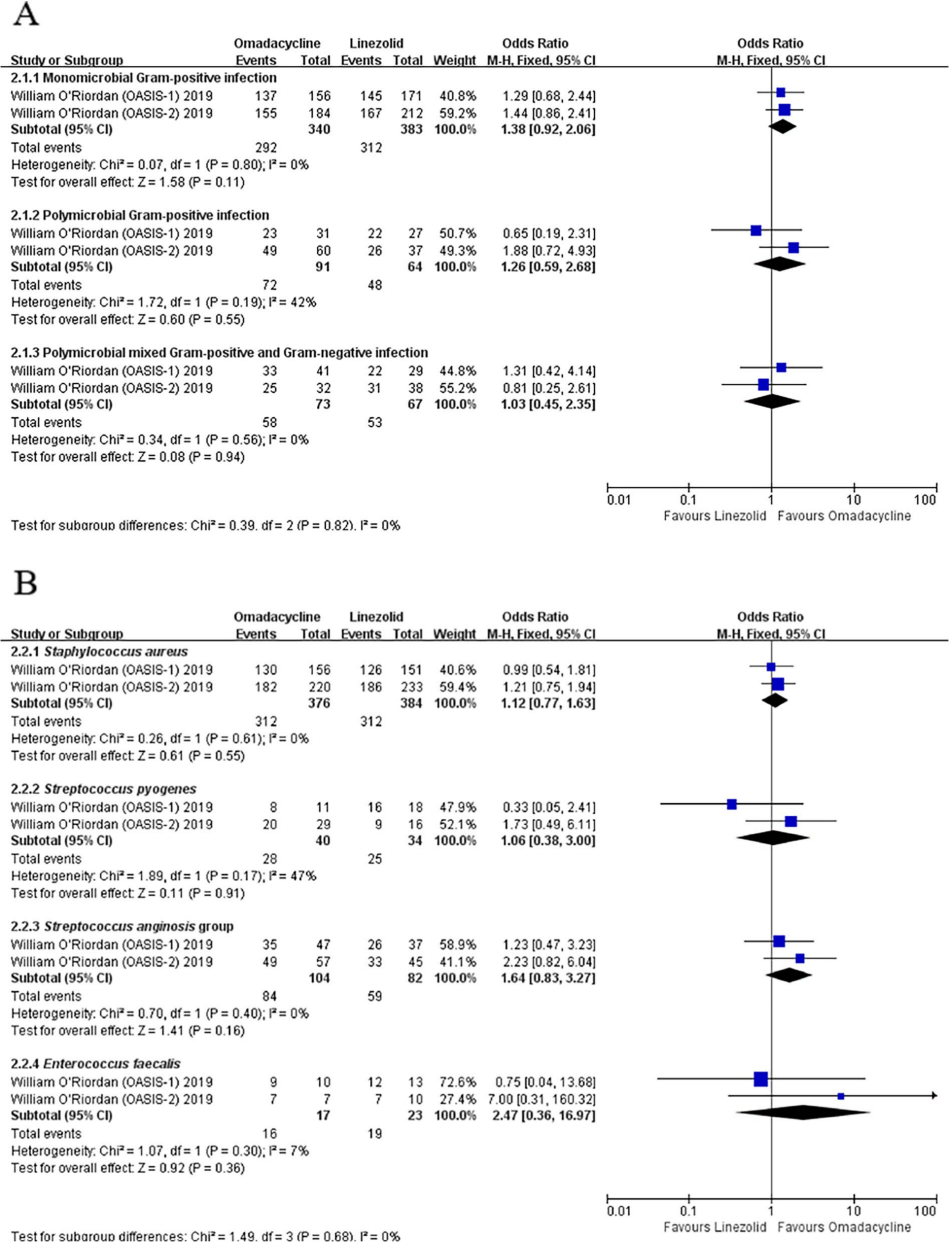
Microbiological response of OMC and LZD in the micro-MITT population. **A** Microbiological response in different types of pathogenic bacteria. **B** Microbiological response with common Gram-positive bacteria

The microbiological response of OMC in the micro-MITT population at PTE was not inferior to LZD among patients with monomicrobial Gram-positive infection (OR: 1.38, 95% Cl: [0.92, 2.06], *P* = 0.11), polymicrobial Gram-positive infection (OR: 1.26, 95% Cl: [0.59, 2.68], *P* = 0.55), and polymicrobial mixed Gram-positive and Gram-negative infection (OR: 1.03, 95% Cl: [0.45, 2.35], *P* = 0.94) (Fig. 5A). Next, we compared the ability of OMC and LZD to eradicate common Gram-positive bacteria that caused cSSTIs. The results suggested that OMC was not inferior to LZD in eradicating Gram-positive pathogens (*S. aureus*: OR: 1.12, 95% Cl: [0.77, 1.63], *P* = 0.55; *S. pyogenes*: OR: 1.06, 95% Cl: [0.38, 3.00], *P* = 0.91; *S. anginosus* group: OR:1.64, 95% Cl: [0.83, 3.27], *P* = 0.16; *E. faecalis*: OR: 2.47, 95% Cl: [0.36, 16.97], *P* = 0.36) (Fig. 5B).

### Safety

**Fig. 6:**
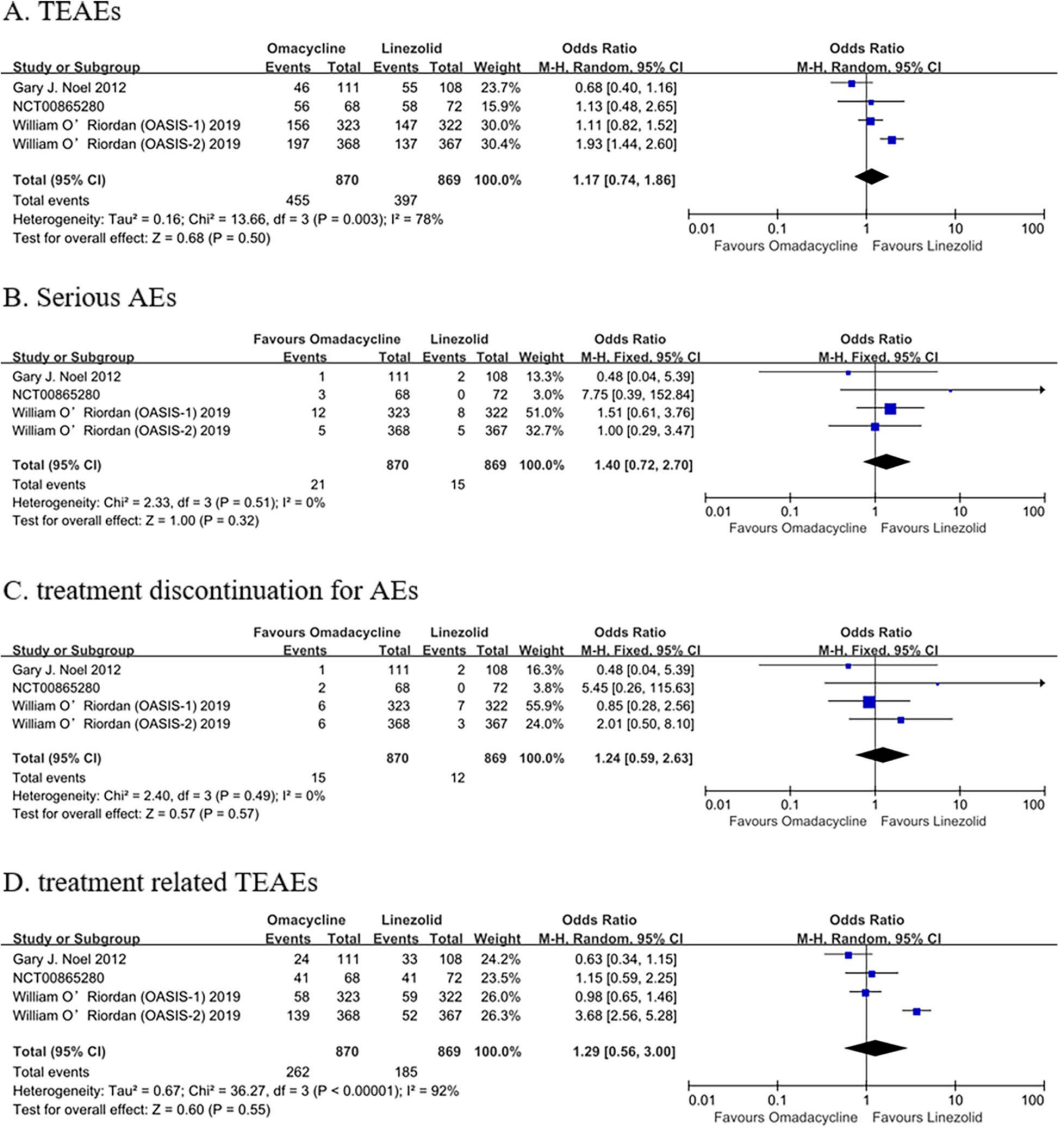
Overall AEs of OMC and LZD

**Fig. 7:**
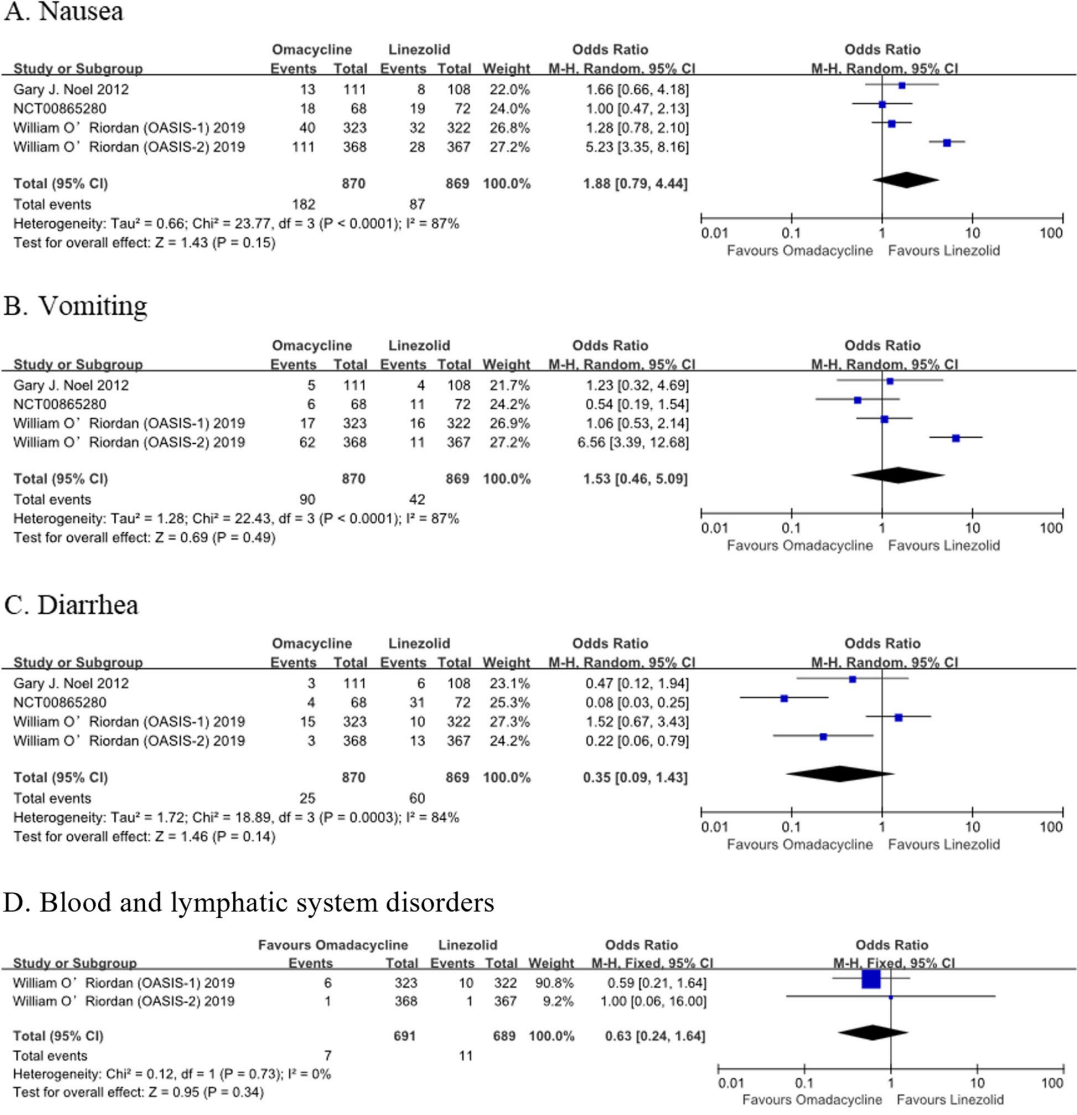
The incidence of common TEAEs of OMC and LZD

There was no significant difference in treatment-emergent adverse events (TEAEs) (OR: 1.17, 95% Cl: [0.74, 1.86], *P* = 0.50), serious AEs (OR: 1.40, 95% Cl: [0.72, 2.70], *P* = 0.32), treatment discontinuation for AEs (OR: 1.24, 95% Cl: [0.59, 2.63], *P* = 0.57), and treatment related TEAEs (OR: 1.29, 95% Cl: [0.56, 3.00], *P* = 0.55) between OMC and LZD (Fig. 6). In addition, no significant difference was found in nausea (OR: 1.88, 95% Cl: [0.79, 4.44], *P* = 0.15), vomiting (OR: 1.53, 95% Cl: [0.46, 5.09], *P* = 0.49), diarrhea (OR: 0.35, 95% Cl: [0.09, 1.43], *P* = 0.14), and blood and lymphatic system disorders (OR: 0.63, 95% Cl: [0.24, 1.64], *P* = 0.34) (Fig. 7).

**Fig. 8:**
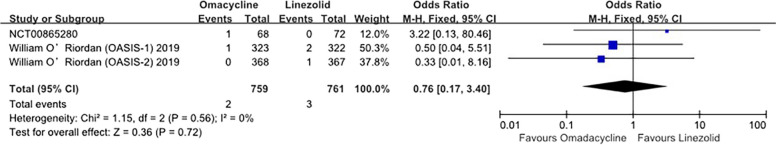
Mortality rate of OMC and LZD

In terms of mortality, there was no significant difference between OMC and LZD (OR: 0.76, 95% Cl: [0.17, 3.40], *P* = 0.72) (Fig. 8).

## Discussion

Our meta-analysis consisting of four RCTs showed that the clinical efficacy of OMC was as good as LZD. A previous meta-analysis has evaluated the efficacy and safety of OMC in the treatment of acute bacterial infection, which includes three studies on cSSTIs and one study on CABP. The results show that the clinical efficacy of OMC is numerically higher compared with the comparator antibiotics, and OMC shows similar clinical efficacy to LZD in treating cSSTIs [29]. However, the study did not appear to unify the criteria for the MITT and CE populations included in the study (two studies excluded patients with Gram-negative bacteria only; one study did not and added aztreonam against Gram-negative bacteria). After intravenous injection of OMC in rats, the skin-to-blood concentration ratio is increased by 6.6 times [30]. In healthy subjects, the steady-state distribution volumes of OMC are 190 ~ 204 L [17, 18], and the plasma protein binding rate is about 21% [10]. In contrast, the steady-state distribution volumes of LZD are 40 ~ 50 L [31], and the plasma protein binding rate is about 31% and has poor penetration into adipose tissue [32]. These findings may indicate that OMC has a high rate of tissue penetration than LZD.

The microbiological response of OMC was numerically higher than LZD, which might be related to its lower MIC. *S. aureus* is the most common pathogen causing cSSTIs, accounting for 81% of isolated pathogens [4]. Compared with LZD, OMC has a lower MIC against common Gram-positive bacteria in vitro. For example, for *S. aureus* (including MRSA and MSSA), *S. pyogenes*, and *E. faecalis*, the MICs are ≤ 0.015 ~ 2 mg/L for OMC and ≤ 0.12 ~ 8 for LZD [33]. The tissue distribution concentration of OMC and LZD is 378 mg/L and 15.5 mg/L, respectively [30, 34]. Free concentrations of OMC and LZD at the skin and soft tissue sites could cover common Gram-positive pathogen bacteria.

Although Gram-positive bacteria are the most common pathogens, Gram-negative bacteria or mixed infections still account for about 12 ~ 21% of severe skin infections [6, 35]. Among Gram-negative pathogens, *Enterobacteriaceae* is the most common isolated bacteria [36]. OMC shows activity against Gram-negative bacteria in vitro, such as *Escherichia coli* (*E. coli*), *Klebsiella pneumoniae* (*K. pneumoniae*), and *Haemophilus influenzae*, with MIC_50/90_ values of 0.5/2, 1/4, and 0.5/1 mg/L, respectively [33]. In recent years, multidrug-resistant (MDR)/extensively drug-resistant (XDR) bacteria have also been isolated from cSSTIs (such as MDR/XDR *Enterobacteriaceae*) [35]. A multicenter, observational study involving nine patients has found that oral OMC (450 mg loading dose plus 300 mg maintenance dose) is effective against MDR *E. coli* and *K. pneumoniae*-induced bone/joint or intra-abdominal infection [37]. Since LZD is not active against Gram-negative pathogens [38, 39], patients with sole Gram-negative pathogenic bacterial infections were excluded from RCTs. Therefore, we could not analyze how effective OMC was in treating cSSTIs caused by Gram-negative pathogens. In this analysis that integrated OASIS-1 and OASIS-2, the microbiological response of mixed infection with Gram-negative strains of OMC and LZD was similar in the micro-MITT population at PTE, and this finding might be attributed to the small number of cases. Considering that OMC is effective against Gram-negative bacteria or mixed infections, it might be preferred for treating cSSTIs.

Regarding safety, the incidence of adverse drug reactions in OMC was similar to that in LZD, and most of them were transient AEs. Unsurprisingly, since OMC is structurally similar to tetracycline antibiotics, the most common AEs are gastrointestinal events [40]. It was observed that the incidence of nausea and vomiting in the OMC group was slightly higher compared with the LZD group in this meta-analysis. Some studies have shown that the gastrointestinal AEs of OMC are dose-dependent in healthy and diseased subjects [41, 42]. A higher incidence of gastrointestinal AEs in the OASIS-2 study with a higher dosage applied is also observed. Studies have shown that oral OMC after meals can reduce gastrointestinal AEs. However, compared with fasting, taking meals 2 to 4 h before the administration will reduce the bioavailability of OMC [43]. Therefore, to obtain an excellent therapeutic effect, oral OMC should be taken on a fasting state and avoided in combination with dairy products. Some AEs associated with the long-term use of LZD include myelosuppression, peripheral and optic neuropathy, serotonin syndrome, and so on [44]. Only a trend of a higher number of AEs in the hematological system was observed in the present meta-analysis (1.60% in LZD vs. 1.01% in OMC). This finding might be related to the short duration of treatment (the treatment time was 7 ~ 14 days). Studies have shown that the hematological system response of LZD is increased with the prolongation of medication time (treatment duration ≤ 14 days reported 1.9%; 5.1% for 15 ~ 28 days; 7.4% for > 28 days) [45]. Although the recommended duration of cSSTIs treatment is 7 to 10 days, the treatment time should be extended if the infection is not improved during treatment [46]. Therefore, it is still necessary to pay attention to the hematological toxicity of LZD.

This study has several limitations. Firstly, the study failed to demonstrate that OMC was superior to LZD against Gram-positive infection. The ME population included a CE population with at least one Gram-positive pathogen at baseline, but there was no significant difference between OMC and LZD. Moreover, although no restrictions were set on the types of control drugs, only LZD was included in this meta-analysis. Comparisons between OMC and other antibiotics, such as vancomycin, for the treatment of cSSTIs could not be evaluated here. Lastly, because LZD was ineffective against Gram-negative bacteria, we could not assess the efficacy of OMC against infections caused by Gram-negative bacteria.

## Conclusions

This meta-analysis showed that OMC was as good as LZD regarding clinical efficacy and microbiological response, and has a similar safety profile. Therefore, OMC might be a promising option for treating cSSTIs in adult patients. However, we need to further study the hepatorenal impact and low immune function populations before applying OMC in Gram-negative or mixed cSSTIs.

## Data Availability

The data sets generated during and/ or analyzed during the current study are available from the corresponding author on reasonable request.
